# ‘The world is full of magic things, patiently waiting for our senses to grow sharper’ (WB Yeats): enhancing resilience among deaf young people in South Africa through photography and filmmaking

**DOI:** 10.1136/medhum-2019-011661

**Published:** 2020-01-20

**Authors:** Alys Young, Lorenzo Ferrarini, Andrew Irving, Claudine Storbeck, Robyn Swannack, Alexandra Tomkins, Shirley Wilson

**Affiliations:** 1 SORD (Social Research with Deaf People), School of Health Sciences, University of Manchester, Manchester, UK; 2 GCVA (Granada Centre for Visual Anthropology), University of Manchester, Manchester, UK; 3 CDS (Centre for Deaf Studies), University of the Witwatersrand, Johannesburg, South Africa; 4 NSPCC (National Society for the Prevention of Cruelty to Children), London, UK

**Keywords:** social anthropology

## Abstract

This article concerns deaf children and young people living in South Africa who are South African Sign Language users and who participated in an interdisciplinary research project using the medium of teaching film and photography with the goal of enhancing resilience. Specifically, this paper explores three questions that emerged from the deaf young people’s experience and involvement with the project: (i) What is disclosed about deaf young people’s worldmaking through the filmic and photographic modality? (ii) What specific impacts do deaf young people’s ontologically visual habitations of the world have on the production of their film/photographic works? (iii) How does deaf young people’s visual, embodied praxis through film and photography enable resilience? The presentation of findings and related theoretical discussion is organised around three key themes: (i) ‘writing’ into reality through photographic practice, (ii) filmmaking as embodied emotional praxis and (iii) enhancing resilience through visual methodologies. The discussion is interspersed with examples of the young people’s own work.

## Introduction

This article concerns deaf young people in South Africa, the vast majority of whom were South African Sign Language (SASL) users, and their engagement with a research project that used film and photography as the media of research methods and outputs. The overall focus of the project concerned enhancing resilience and was built around specific objectives of promoting positive aspirations, interventions to support emotional literacy and child/youth safeguarding. This article does not report the results of that work, if by that we mean the extent to which original project aims and objectives were met. Details of that will be available in forthcoming publications. Instead, this article explores theoretically three issues that emerged for the research team through the pursuit of the research project and which arose through deaf young people’s engagement with the medium of the project—film and photography. They address how this modality discloses deaf young people’s world making and conversely how the ontologically visual habitations of deaf young people impact on film/photographic work and what these relations between visual ontologies and visual outputs might tell us about enhancing resilience in deaf youth. First, by way of orientation, we set out transparently the standpoint from which we began our work as this was fundamental to decisions made about how we pursued the project and the theoretical reflections and arguments we offer.

### Perspective

Deafness does not just affect hearing; it fundamentally affects vision.1 Whether the visual is understood as an enhanced sense in the absence of full hearing,2 or a linguistic medium, including sign language and other forms of visual communication,3 deafness involves an alternative visual understanding of and relationship with the world. Cognitive science and educational research are increasingly demonstrating a visual bias and visual strength among deaf learners,4, 5 derived in part from the habitual and ongoing deployment of visual forms of knowing and understanding, regardless of whether one speaks or signs.6 From a deaf cultural perspective, a strong visual orientation to the world is considered central to deaf cultural identity.7, 8 Commonly used phrases such as ‘people of the eye’9 emphasise that to be deaf elicits a different visual and ontological relationship with the world. *Being deaf* is to experience and have presence in the world in ways that are not predicated on hearing and is generative of different kinds of knowledge.10, 11 Consequently to be deaf encompasses specific ontological orientations to the world. By this we mean, after Heidegger,12 that our everyday involvement in the world is shaped through our practical engagement with the existences of things, states and people (ontics) with reference to *how* they are present and disclosed to us through use, activity, purpose and experience in ways that build an understanding of their distinct properties and character (ontology). It is through use, proximity and practical familiarity—as experienced ontically through the totality of our relationships to and ongoing activity in the world—that reflexive and theoretical understanding develop and are made possible, thereby forming the foundational conditions for any specific and systematic (ie, ontological) inquiry into the nature of things. ‘What the one thing is will depend on the other thing, on what it is’ 13 (269).

It is essential to recognise that assertions that being deaf offers a *changed* sensory orientation to the world, presupposes and reinforces a phonocentric point of view in which the primacy of sound and hearing is construed as the normative base from which all else is an aberration.14, 15 It is an assumption memorably described by Derrida as ‘the most original and powerful ethnocentrism’16 (70). Our attempt to move away from this positioning is to argue that recognising and engaging with how deaf people visually experience and portray the world is contributory to the sum of the full capacity of what it is to be human and therefore of saliency to all. In an inversion of Alexander Graham Bell’s17 eugenic designation of ‘a deaf variety of the human race,’ Bahan7 argues deaf people should more readily be considered a ‘visual variety of the human race,’ a concept taken up by others to focus attention on ‘deaf gain.’18, 19 The phrase does not just refer to the rarely considered advantages of being deaf on an individual level but also the societal gains attendant on the recognition of deaf people’s unique ontological and epistemological credentials. What may all people learn and gain from the lived experience of deaf people(s) and how may society as a whole be enhanced and grow from such engagement?

It is important to begin by setting out this perspective because it was fundamental to how we carried out the project we will describe and discuss below. Our key starting points that influenced the execution and analysis of resultant work were:

- the positioning of deaf people as ontologically visual
- possessing of visual strengths (cognitively, epistemologically and expressively)
- whose lived experience is equal-and-different, not impaired, to those who hear
- and whose fundamental visual orientation releases potential to portray the world in ways that are transformative for others.

We now describe briefly the context of the overarching project and introduce the team involved.

## Project context

### Project focus

‘Enhancing resilient deaf youth in South Africa’ was a joint-funded project by the Arts and Humanities Research Council and Medical Research Council (Reference: AH/R00580X/1) under the Global Challenges Research Fund call for public health networks. Jointly lead by Young (SORD, University of Manchester, UK), Irving (Granada Centre for Visual Anthropology, University of Manchester, UK) and Storbeck (Centre for Deaf Studies, University of the Witwatersrand, South Africa), the project worked alongside two non-governmental organisations also based in South Africa offering early intervention and parent support services, HI HOPES and THRIVE, and six schools for the deaf, four in KwaZulu-Natal and two in Gauteng. Through a creative interdisciplinary collaboration between visual anthropology, social science and deaf studies, the project set out to enhance resilience among deaf youth in South Africa through the medium of film and photography based around a series of interactive workshops with deaf young people.

We deliberately use the term ‘enhance’ rather than ‘build’ resilience because from the start we acknowledge that deaf young people already possess considerable resilience when negotiating the social and economic context in which they are growing up (see below). We conceptualise resilience as an *interaction* between psychological traits such as good self-esteem and effective coping strategies, and the social context.20 Social context, in line with Causal Agency Theory21 is understood in terms of resources and/or affordances, at individual and societal levels (such as quality education) which enable the agentic self. It also encompasses those forces which limit and deny the development of a self-determined life through processes such as social stigma, economic inequality and structural barriers to access to resources (health, education, technology). We followed the definition of resilience with respect to deaf children offered by Young, Green and Rogers20 (52): ‘… *the successful navigation of being deaf* in a world that faces (deaf children) with countless daily hassles and which may commonly deny, disable or exclude them’. Although originally defined in relation to technologically and economically advantaged countries, the emphasis on navigation, the realisation of the deaf self and the social forces that may act to enable or deny such development is applicable across social and economic contexts. We return to this framework in considering the processes and outputs of the work described below.

### Country context

The project’s three themes of raising aspirations, developing emotional literacy and child/youth safeguarding were highly salient in a country context already subject to high levels of violence and abuse directed at children, and for which deaf children are further at risk. According to police statistics, in the year 2011–2012 alone, 50 688 children were victims of violent crime in South Africa including 793 who were murdered, 758 who were victims of attempted murder and 25 682 who were victims of sexual offences.22 The national prevalence of neglect and ill-treatment of children is reported as 8.1% with a range between 4.2 and 17.2 dependent on Province.22

In all, 6200 deaf babies are born annually in South Africa, 90% of whom will not be early identified nor receive rapid follow-up early intervention support services23 which is now the global standard for best practice to promote optimum linguistic, cognitive and social development.24 Many more children become deaf as a consequence of childhood illnesses. There are at least 43 schools for the deaf addressing primary and secondary education (day and residential) in South Africa25 offering education through SASL and/or spoken language. Significantly, not all deaf children can access or receive an education and it unknown how many deaf children do not go to school. Migrant parents may be reluctant to bring their deaf children to attention because to do so would expose their own illegal citizenship/residence status.26 For those with South African citizenship on low incomes, a Care Dependency Grant is offered which covers the cost of deaf children being educated in special schools. However, it does not enable choice of educationally better deaf schools whose fees are prohibitive and not covered by the allowance. For those without citizenship, no allowance is available to support the costs of raising a deaf child in South Africa. South Africa is defined as an Upper Middle Income Country on the DAC List 27 of countries eligible to receive Overseas Development Assistance. 55% of South Africa’s population live in poverty and this is disproportionately distributed between ‘racial’ groups, with 93% of those living in poverty being classified, in line with the South African Census classifications, as Black.28 29 Only 16% of South Africans have private health insurance30 which would cover, at least partially, the costs of hearing technologies such as hearing aids and until recently would not cover at all any costs towards cochlear implants (CIs). CIs are sporadically rather than universally available through charitable foundations for those without private means. Access to spoken language acquisition for those with significant hearing losses is, therefore, severely impeded. At the same time, access to early language role models of SASL and opportunities for natural sign language development are also delayed31 for the over 90% of deaf children who will have hearing speaking parents. In citing this figure, we follow the commonly cited US reference32 but acknowledge that there is no reliable epidemiological evidence to support or refute its validity for South Africa.

### The team

The team comprised colleagues from South Africa and the UK, deaf SASL users resident in South Africa and deaf British Sign Language (BSL) users resident in the UK, and hearing academics from both SA and UK some of whom were experienced signers with many years’ experience working with deaf children/adults, some of whom were not. The visual anthropology component of the project was led by Irving and Ferrarini from the UK and two masters level qualified visual anthropologists, one UK based (Tomkins) and one South Africa based (Swannack). With the exception of Swannack, who is a native SASL user from a deaf family, the anthropology component of the team was hearing with little or no prior knowledge of working with deaf children or alongside deaf colleagues. The deaf studies/social science component of the project was led by Storbeck (SA) and Young (UK) with the addition of one post-doctorally qualified social scientist (Rogers) and masters-level qualified social scientist (Wilson) both of whom were deaf BSL users. All members of the deaf studies component of team were fluent sign language users in either SASL, BSL or both. Additionally, the team employed a deaf SASL filmmaker based in South Africa (Mbazima), a deaf SASL teaching assistant (Mangele), a project co-coordinator who was a parent of a deaf child growing up in South Africa (Clelland) and eight parent facilitators all of whom had direct experience of raising a deaf child in South Africa. A range of SASL interpreters worked with the team when required but around half of the teaching and facilitation with the children occurred directly through SASL via the fluent SASL using team members. The interpreters had never previously worked within a visual anthropological approach, and with hindsight greater attention to this fact and training would have been helpful. Shortages of interpreters meant that it was challenging to maintain the same SASL interpreting team for the whole project on all days of activity which was not optimal.

We set out these identity characteristics of the team members to demonstrate that this was a highly complex interdisciplinary, cross-cultural (SA/UK and deaf/hearing), multi-linguistic, ethnically diverse team. We were strongly aware of the history of double colonialism in the context in which we were working, taht is, in terms of South African history and apartheid33, 34 but equally the widespread prevalence of colonial attitudes and practices among hearing people toward deaf signers.35 Consequently, we sought to ensure that both the immediately obvious characteristics of the team members and our values and praxis as a collaborative team demonstrated our commitment to combatting that historical legacy. The challenges involved within the interdisciplinary, cross-cultural team are examined in a forthcoming paper.

## Methods

The overarching project’s aims were as follows: (a) Through a child and community authored method, to involve deaf children/youth in the making and production of a series of ‘this is me—this is my future’ films; (b) to support the development of parent–child socio-emotional interactions; and (c) to develop a series of ‘growing up and keeping safe’ films aimed specifically at deaf young people. We set out below the anthropological approach and visual methods adopted in pursuit of these project aims before identifying the emergent research questions that arose from this process and which shape the theoretical discussion that follows.

### Methods

Anthropology is a foundational discipline in the use of visual methods in general and film and photography in particular.36–38 Its leadership and legacy are acknowledged by deaf studies in its more recent adoption of visual methods strategies.39–42

At the outset, the use of photography and film in our study was conceptualised as both a skill to be taught to the deaf children and young people with whom we would work, and an appropriate and effective pedagogical mechanism for exploring issues, topics and questions that other approaches might struggle to engage children and young people with. Photography and film are particular modes of embodied, performative and collaborative activity that create contexts for collective learning, exploration and expression. As such, the young people’s introduction to and use of the camera involves a process of attunement and attention that establishes a new awareness of and relationship between people, their bodies and their surroundings. The intersubjective realm that emerges through composing one’s body in relation to, or looking through, the camera, creates a new learning and pedagogical environment for the generation of thought, action and knowledge, and in doing so becomes a catalyst for creative dialogue and imaginative possibility that might not typically be generated in classroom contexts.

Consequently, we regarded the processes of learning and practise with which the young people engaged and explored their surroundings to be of as much importance in fulfilling the educational aims of the project as the outputs that the young people might produce. This was because we anticipated elements of the process of developing a range of new skills based around the exercises the young people would do (see below), would reveal insights into how they viewed their world for example, through how they might be planning their compositions and working together, as well as choices about how they might want to portray their experience to others (whether explicitly or implicitly). We were not setting out to use the visual medium to elicit data in a process in which we were the researchers and the young people were the research subjects. Nor could our work be described as cooperative enquiry. Instead, a pedagogical approach based on practice-based participation, guided learning, play and improvisation were combined to create a learning context to reveal, as well as generate, understandings that may otherwise have remained unarticulated. Jean Rouch43 actively employed such techniques when using film to elicit the realms of thought, emotion, memory and imaginative possibility that are inherent within the children’s embodied life situation and circumstances. This not only recalls Langer’s idea that ‘most new discoveries are suddenly seen things that were always there’ 44 (8) but also involves an existential process in which play facilitated an opportunity for the children to respond creatively and imaginatively to opportunities made possible by encounters with new persons and technologies.

### The skills teaching component and approach

In broad terms, the teaching component of the input was designed to take the learners through tasks of gradually increasing complexity in terms of still and moving image creation as a means to grasp both core technical knowledge about how to use a camera and core compositional elements in the creation of a good quality image or short film. In schematic terms, see table 1.

**Table 1:** Summary of skills teaching and composition techniques

Increasing complexity						
	Single portrait photograph	Modified multiple portrait photographs	Photographs in narrative sequence (beginning, middle, end)	Simple film sequence based on three-part photo story	Film story based on outline script that has been given	Independently scripted film story
**Technical skills taught/practised**	Camera safety & how to hold a camera Manual focus Zoom	Close up (CU), medium shot M, long shot (L) Establishing shots	Combining (CU), M and L shots to create sequences, variety and action Montage	Stable camera movement Tripod versus handheld Filming CU, M, L shots Panning & tilting Length & duration of each shot	Combining a variety of shots, for example: close up, medium shot, long shot, pan, tilt How to shoot an interview/conversation	Using all technical skills taught/practised
**Composition techniques taught/practised**	Finding your focal point Context-defining subject and backgr-ound Lighting exposure	Perspective—introducing camera angles Framing of face, half- body & whole body Showing character, emotion and capturing body movement.	Perspective—using camera angles for storytelling effect Framing—finding the frame within a frame	Storyboard Thinking in shots & scenes to create a sequence Context—finding your image and adjusting to suit your scene	Building and resolving tension and emotions through the shots used	Using all composition techniques taught/practiced
REINFORCEMENT OF LEARNING THROUGH PEER FEEDBACK Watching together each other’s films and commenting on them to the whole group Expressing preferences and alternatives when watching back the films Using the films to identify techniques that had been used and whether they were effective Re-telling (re-signing) the narrative sequences to the group as a means of sense-checking						

The teaching delivery component took the form of an interactive whole group workshop to describe and demonstrate the task, followed by small group work to reinforce the aim of the task, allow for clarification, support the ideas young people wanted to try out (through encouragement and discussion rather than direction), and specific hands-on technical skills teaching. This group-based approach was culturally coherent with preferences for learning among sign language users. The young people used the contexts of their own school environment to take pictures and later on to develop their short films as it was not practical, and in some cases unsafe, for them to do the work outside of the school boundaries. The working method included participants coming back together as a whole group to view each other’s pictures and films. This provided the opportunity for them collectively to develop critical skills through a facilitated peer discussion of what they liked and why, and what could be improved. In later stages of the work as the young people became more skilled in group critical feedback which of itself was an important learning outcome in relation to understanding visual, aesthetic and representational possibilities and choices. A key objective was to facilitate the development of critical and reflexive skills, which are more readily acquired and understood in terms of the young people’s embodied visual capacities than through other mediums, and whose knowledge can then be transferred and applied to different areas of learning and life. Looking at and giving attention to the world through the camera and its creative outputs becomes a shared basis for the development of collective critical skills and discussion. We also used these sessions to start to explore personal and shared emotional responses to images and intended meanings in portrayal and choice of image, shot, scene and sequence. These processes of formative feedback have for a long time been part of the practice-based filmmaking pedagogy of the Granada Centre for Visual Anthropology45 and are meant to develop a critical understanding of the possibilities and constraints of different visual and sensory media instead of providing fixed rules or abstract general principles.

To avoid distraction, none of the group discussions or small group work was film-recorded ‘as data’. Instead, members of the team were asked to keep a reflective diary as field notes. All session plans and structures of tasks used were recorded alongside the young people’s outputs. This provided a basis for ongoing learning by the team in considering what approaches were effective and how they could be improved. It is the memories and reflections of the team participants and some of their notes on which we largely draw in this paper in its post-hoc reflexive method.

The workshops, generation of photographic and film outputs, the identifiable faces of participants on the outputs and permission for the wider use and distribution was approved through the Human Research Ethics Committee (Education), University of the Witwatersrand, South Africa, the Department of Education, KwaZulu Natal, South Africa and the University of Manchester, UK research ethics committee. Additionally, permission was gained for participation from all children/young peoples’ parents or guardians, from each school head teacher and most importantly from the children and young people themselves.

### Patient and public involvement

The first ideas for this project were discussed with parents of deaf children in South Africa through our partnership with parent-led organisation THRIVE who also contributed to writing the initial grant application. The project team included a parent of a deaf child in South Africa as the project delivery manager directly liaising with the deaf schools involved. School teachers within the contexts in which we worked offered suggested amendments and ideas between the first and second phases of project delivery. The young people involved have and are actively shaping the dissemination of the projects outputs through their continued engagement with photography and filmmaking including co-delivering online and in person exhibitions of their work.

### Participants

In total, the project worked in six deaf schools, two in Gauteng and four in KwaZulu-Natal over 2-month and 1-month periods. In most instances, the schools benefited from two workshops, each on average 4 days long. A total of 72 deaf children/young people participated, ranging in age from 8 to 22 years old. Common to all schools in the project was a primary use of SASL. Very few children had any listening technology such as hearing aids or CIs and even when they did they generally preferred to communicate in SASL as it was the most inclusive medium with respect to their peers. Based on school data and our own observations and interactions, very few participants had age appropriate language in any language and many were severely language delayed.

### Emergent research questions

The following research questions emerged through the practice and products of deaf young people’s engagement with the project’s approach and methods, rather than being the questions that initially framed the project. The questions raised and which frame our discussion were:

- What is disclosed about deaf young people’s worldmaking through the filmic and photographic modality?
- What specific impacts do deaf young people’s ontologically visual habitations of the world have on the production of their film/photographic works?
- How does deaf young people’s visual, embodied praxis through film and photography enable resilience?

By worldmaking, in this context, we refer to the notion that individually and collectively ‘we make our universe out of experience by acts of thought and will’46 (137). Furthermore, such worldmaking is inseparable from the character of the recursive relationship between the ontic and the ontological, whereby our bodily comportment, motor skills and activity already incorporate within them a relational understanding and specific knowledge of the world; in this case, the visual and cultural orientation of the deaf young people.

## Presentation of findings and discussion

Our presentation of findings takes the form of three discussions in which we reflect on concerns that speak to the emergent research questions we had identified. We intersperse a theoretical discussion with examples drawn from the young people’s work so that we are able to show transparently why the themes we discuss were prompted by what we as researchers saw and experienced through the young people’s engagement. We are not presenting a post-hoc analysis of the young people’s photographic/film work, nor are we presenting their words/discussion about their work. We are using examples of their work and our reflections on it as a vehicle to expand on the three research questions that this engagement prompted for us. The discussion sections below do not directly map on to these three questions as some of their material crosses over and between these questions. They are organised into writing into reality through photographic practice; filmmaking as embodied emotional praxis; and enhancing resilience through visual methodologies.

### ‘Writing’ into reality through photographic practice

In a discussion of ‘what ontologies do in deaf worlds’, Friedner11 remarks that ontology is fundamentally concerned with how worldmaking happens. Therefore what a person understands about their world (and also what is misunderstood or unknown) is inseparable from how subject and object is conceived, and also by extension how referentiality is engendered and maintained, although her argument does not extend that far. In the case of deaf signers, she argues that it is communication barriers that are fundamental to deaf ontologies in that they hinder access to formal and incidental knowledge and circumscribe the possibilities of social exchanges through which relational knowledge and understanding is produced. Her argument traces cultural practices of checking understanding and meaning negotiation among deaf peoples where it is accepted that not understanding is a commonly occurring experience which deaf people combat through collective, social, communicative strategies.

However, observations such as this on the origin and nature of aspects of deaf ontologies do not address the mechanisms that serve, in Foucauldian terms,47 to consistently (re)create the subjectivities to which a people may be bound. For example, in South Africa economically imposed barriers to quality education, among other socio-structural factors including discrimination, that deny the opportunity for good literacy for deaf people, the possession of which would also combat states of misunderstanding and exclusion. Just because something is does not mean it has to be. However, as Povinelli48 (459) argues, for ‘the otherwise’ to occur (for potentiality to be enacted) requires *the conditions* in which the performativity of self may be possible; individual volition is not sufficient. In providing young deaf people with the skills and practical resources for photography, we were providing such conditions for exploration of subject and object grounded in and inseparable from deaf young people’s visual embodied experience (their ontological potentiality) *and* providing a means to communicate that alterity to others in the created image.

In so doing, we built on experiences of studying image-making practices in situations where visual anthropologists provided guidance and training. From Worth and Adair’s49 pioneering work with young Navajo filmmakers we draw an approach to image-making not just as representations of reality but which are also statements on or generative of reality, part of a semiotic system distinct from verbal language.50 During the 1980s and 1990s, multiple experiences with indigenous and minority media, chiefly in Canada, Brazil and Australia51–54 helped us in considering the political aspects of enabling deaf youth to tell deaf stories. But in contrast to all these experiences of the past, we were not looking for distinctly deaf ways of making images to be revealed through our workshops, well aware that deaf youths, in South Africa as elsewhere, are experienced consumers of global visual cultures, and that our own teaching praxis and technologies influenced decisively the results. We approached instead the use of the camera as an embodied visual technology that provided a material referentiality that was at one with the ontological orientation of those behind the camera.

This materiality of image was important because signed languages are non-orthographic; a fact that has consistently denied the possibility of deaf peoples *writing into reality* their own worlds for others to understand and be influenced by.55–57 Although the written word has not always been the means to create and control social discourse and narrative, as the mediaeval iconography of the Catholic Church in Europe demonstrates for example,58 it has in modern history been the dominant medium. Consequently, vestiges of deaf lives and his/herstories have been more usually written by hearing people with easy command of the written word and thus through hearing people’s ontological normalcies.59 Through photography, the project was able to provide the medium and means for creating, not just documenting, everyday reality on deaf young people’s terms. It was not so much that dominant narratives ‘about’ deaf young people from the hearing majority were resisted, but rather that through the many images produced, the subjugated knowledges of young deaf South Africans (subjugated because of their visual form of knowing and telling) were being released.

The following photographs are just a few of the many hundreds produced. All were planned, enacted and photographed by deaf young people themselves. In recognition of the polyphonic nature of sense-giving,60 we note the narratives below are an interpretation by the authorial team. Although influenced by discussions at the time with the young people, they are not intended as a representation of the young people’s views or interpretations. Rather, we wished transparently to demonstrate our own understanding and responses to the images as this cannot be disconnected from how we have theorised the young people’s worldmaking. For sake of validity, our interpretative arcs should be on show too. However, we invite readers to their own meaning making processes in response to their engagement with the images, which will be different and influenced by your own biographies and situated ontologies.

**Figure 1:**
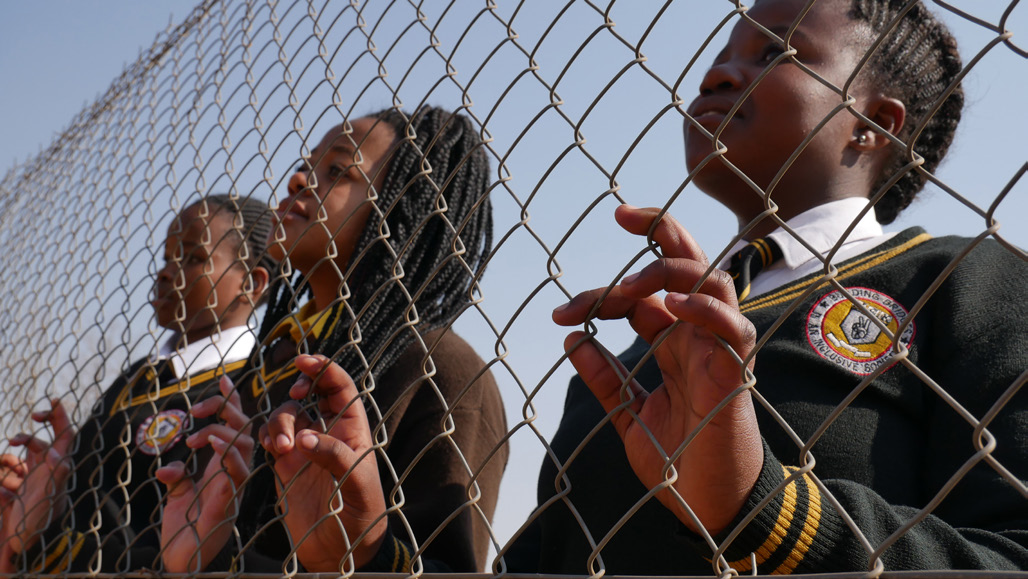
Barriers.

Authorial commentary: ‘Barriers’ was taken in response to the question ‘How would you describe your experience of being deaf in South Africa to a hearing person?’ In this photograph, ‘The Cows’ (each group gave themselves a name) use the fence which surrounds their school to evoke and bring to life the social, economic, educational and linguistic barriers they face on a day-to-day basis. Looking out at a social world from which they are marginalised and excluded, they also look onto a future that has no place for them. But their eyes look up not down and their hands have prominence. Eyes and hands are the channel of communication for signing peoples and their positioning in this image directs the gaze to this source of strength. Looking out through the fence, yes, but also challenging others to look in and to see their defiance. The fence is a barrier also for those who cannot recognise and benefit from their talents and contributions.

**Figure 2:**
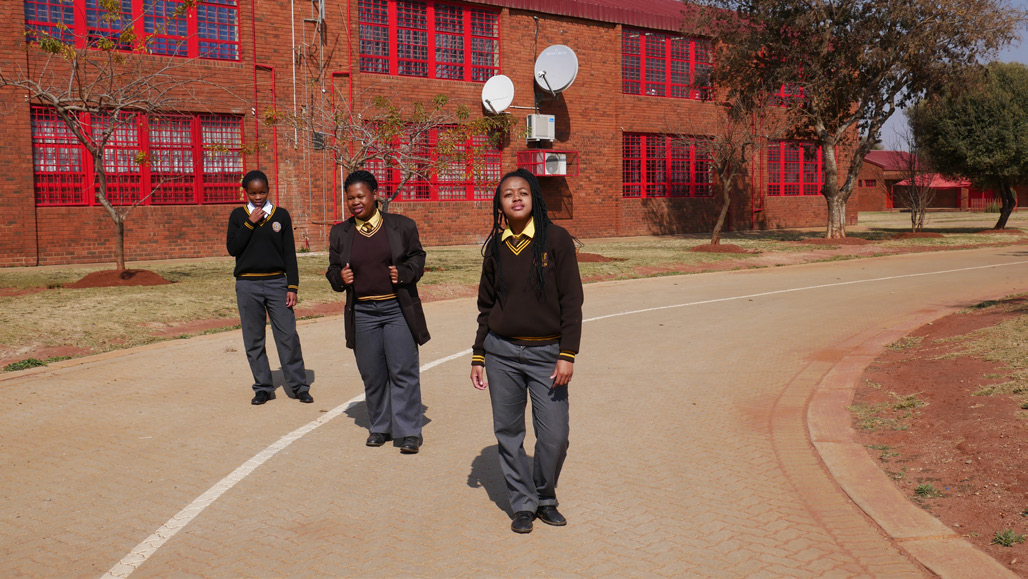
Swag.

Authorial commentary: Swag is also a response to the question ‘How would you describe your experience of being deaf in South Africa to a hearing person?’ It is an image that communicates self-assurance, confidence and solidarity through the arrangement of posture. It is both technically and aesthetically outstanding in its planning and execution: the bodies are precisely arranged in the style of Euclidean or Cartesian perspective and in relation to the sun so the shadows are in alignment. Counterpoint and balance is incorporated into the photograph through the strategic positioning of the three subjects in relation to the line and curve of the road. Everyday life is never straightforward for a deaf signing person, but the bends in the road are creative with possibilities too.

**Figure 3:**
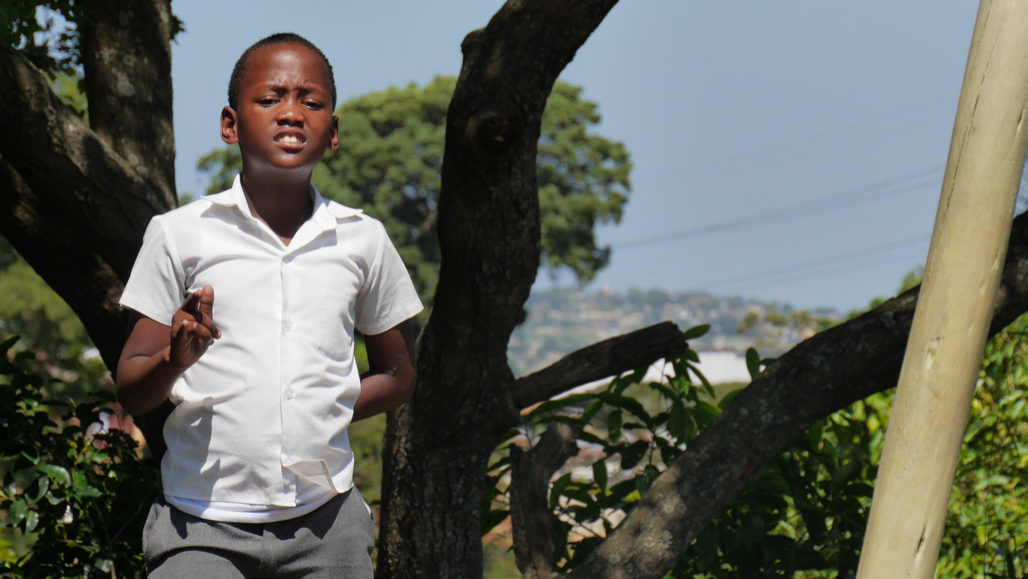
My Friend.

Authorial commentary: Taken in response to an exercise that asked primary school children to represent the emotion of friendship in a single photograph, ‘My Friend’ is remarkable as being the first ever photograph taken by the young photographer. The young photographer, who took this image, only had command of some basic signs. He took a long time in setting up, framing and taking this image and there is clearly a substantial visual intelligence at work in the precise placement and construction of a photograph that evokes the feelings of friendship. Pay close attention to the right hand which says ‘I love you’ back to the photographer, while the left hand, manifest in its absence in the photograph, serves to emphasise the focus on that single message.

**Figure 4:**
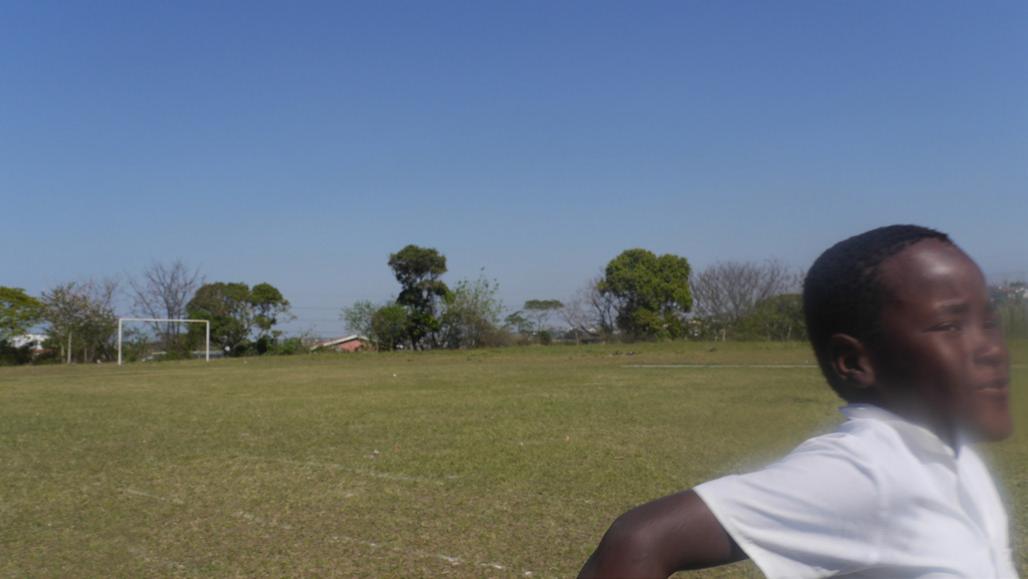
Speed.

Authorial commentary: In response to an exercise that challenged the deaf young people to evoke what it is like to be deaf, ‘Speed’ was taken at a primary school and brings to life the physical and emotional intensity of running. A blur of energy and motion, the placement of the runner and framing are playful, brilliant and atypical in that the vast majority of photographs would simply place the runner on the left of the frame. Here, it as if the runner is about to burst through the frame through sheer speed. This group of children closely associated participation in sports as integral to what it is like to be deaf. Yet, deaf people remain excluded from most mainstream sports events and are usually disallowed from competing professionally with hearing people.

### Filmmaking as embodied emotional praxis

The visual orientation of deaf peoples is only half the story in any discussion of ontology and epistemology; embodiment is of equal and related significance because signed languages are *of* and through the body. Although biomedical and educational discourses are apt to separate language from the person (and the body) in respect of deaf people in distinguishing whether an individual speaks, signs or both (and how well), culturally deaf people do not see it that way.35 The totality of being deaf cannot be thematised into a taxonomy of hearing and language. From a culturally deaf perspective, to be deaf and to sign are indivisible because the embodied self and the expressive self are co-terminus. Language (not just communication) is inscribed on and performed through the body.

Although it may be argued that this is true of all humans in that hearing speaking people are also embodied language users given the organs of articulation are required to have language,61 it is different with deaf signers. This is because the whole body, existing in space, time and context is required to use a language which is four-dimensional; the usual three planes plus the addition of time, for example, handshapes and their movement may be modulated through perseveration and rhythm to indicate intensity.62 The disembodied voice of Beckett’s ‘Not I’63 (213), for example, is an impossibility. Person and language are incorporated (through the body ‘spoken’).15 The embodied nature of experience and the embodied means of its ‘telling’ are enmeshed. In ontological terms, a corporeally produced language both enacts and instantiates at one and the same time. Language, and for that matter culture, is not a generalised abstraction from the particulars of communication or behaviour, it is a ‘concrete universal’64 (445).

This relationship is of a different order to that theorised in mainstream (non-deaf related) work concerning the body as an interactive component in sense making,59 a component of cognition65 or a material inscription of identity(ies).66 This is because the communicating and communicated to elements of the embodied self are language in its complete form; they are not gesture, culturally attributable habits or trace elements of identities memorialised in how the body acts in time and space. They are language and furthermore a visual language closely allied with a visual cognitive habitus. How then might the embodied visual linguistic experience of being deaf influence the creative forms of deaf young people’s filmmaking and what insights might this generate into their world view? From our point of view, the act of filmmaking afforded the opportunity not only to document an experience but rather to enable a reflexive and generative account of lived experience not just in its content but in the terms of its portrayal.

These considerations were evidenced in a film exercise set for one of the groups of young people, designed by the researchers to be radically different from more conventional dialogue, narrative or process-based types of filmmaking. The young people were asked in groups to choose and discuss an emotion they wished to represent and then film three shots to evoke that emotion without using people, the body or dialogue. Instead, they had to encapsulate that emotion through a visual metaphor using and considering the material and other properties of their immediate surroundings.

Behind the setting of this exercise was awareness that a lack early and consistent exposure to emotionally toned language is a common consequence for deaf children growing up without access to sufficient quantity and quality language in their environment, whether that is signed or spoken.67 Emotions in self and others may be felt or observed but they are de-contextualised from the language that ‘speaks’ about them if that language is inaccessible or partially perceived. There is limited exposure and access to the intersubjective communicative experiences which build a repertoire of not just referential terms but also referential experiences through which we come *to recognise what we feel*. Where early language environments are fluent, accessible and communicating, as in the case of the small minority of deaf young people who grow up with deaf parents, these socio-emotional deficits are far less likely to occur. In some cases, feelings experienced have no linguistic referent without which the exploration and/or regulation of one’s emotions and inner life becomes significantly challenging.68 However, the film method and exercise we were proposing required a process of creative and technical visualisation in which it is perfectly possible for unarticulated or inarticulable assemblages of embodied emotion to be rendered into a public, symbolic and expressive form. Lived experience incorporates complex amalgamations of emotion, mood, perception, sensation and interpretation—alongside non-linguistic, imagistic, and non-symbolic modes of thinking and being that operate close to or beyond the threshold of propositional language—but can be evoked through images that have expressive and affective capacities to communicate to others.

For instance, one of the older groups, aged 15–20 years, identified the complex and undulating emotional assemblage that is experienced, for example, during the elongated and enforced waiting for an important piece of information such as the outcome of an exam or a job interview and not knowing when it will arrive. The embodied experience simultaneously encompasses anxiety, uncertainty, desire to know, frustration, wishing they would hurry up, hope and fear, which wax and wane to form a churning and unfolding experience that does not exist in stasis. In describing the complexities and nuances of the emotion to each other in SASL, the productive grammatical properties of the language enabled them to show the emotion in space, handshape, location, movement, facial expression and bodily movement in such a way as to create a common recognition and understanding of the emotion. Although the question remained as to what that emotion could be called, whether in terms of a lexical item in SASL or in its English form. The group settled on *‘frustration’* as the nearest but inadequate name label for the emotion then set out to film and show this emotion in terms of the exercise’s guidelines.

To represent the churning, restless and dynamic qualities of *frustration*, understood as a embodied and lived experience rather than semantic category, the group first collected a large amount of fallen dried leaves and debris that was laying on the ground and assembled it into two large piles. The camera frame was set up so that the leaves were just out of shot, then through a coordinated action the leaves were then repeatedly thrown into the air into the camera’s range to create an ongoing sense of churning and swirling as a dynamic visual metaphor of embodiment and experience. This was followed by leaves still on a tree seen to move in the wind in rhythmic and gentle pattern before turning to a pool of water bordered on one side by low growing plants barely moving but whose movement became visible in contrast to the stillness of the water in which they are reflected (http://deafcamsa.net/projects/emotions/ 2.50–3.19 min).

In another example (http://deafcamsa.net/projects/emotions/ 0.39–1.09 min), a closeup shot of gnarled and interestingly shaped cut tree trunks lying in a disordered way on the ground is followed by a stick being bent and twisted to breaking point by unseen hands before the camera moves our gaze to fine branches of a tree from which green shoots of spring are starting to emerge but they are overshadowed and outnumbered by large fierce thorns with pointed ends. The emotion being portrayed is ‘pain’. What is striking about this creative sequence is how the visual images used also imply bodily sensations as if the distance between feeling pain and seeing pain is foreshortened in this conceptualisation. Alongside the previous example, we would suggest that this feature, we tentatively term ‘corporeally attenuated observation’ is a consequence of the visuo-embodied practice of these deaf young people’s lives wherein they do not communicate through words/signs alone but at the same time inhabit and understand the signs they use through muscle-based activity and the accompanying evocations of personal and collective experience that are performed when describing a sensation or state of being. It is a distinctive example of how words come to life and become incorporated into being, not only through semantic or pragmatic use but in the way that language and communication are grounded in specific uses of the body, personal histories and collective experiences, reinforcing what Foucault and others have observed about the inseparability of subjugated knowledges and practices of the self 47 (461).

### Enhancing resilience through visual methodologies

The two themes we have discussed—writing into reality through photographic practice and filmmaking as embodied emotional praxis—demonstrate the wealth of the material this project has generated as well as the outstanding creative skills of the young people we have encountered, none of whom had ever previously had filmmaking training and in the majority of cases was their first experience of taking photographs. But how does this work we have highlighted speak to the goal of enhancing resilience? We offer three points.

Resilience is not a static, essentialist attribute as if some people are resilient and others are not. Rather it is an interactive and contextualised response capacity in the face of stress, adversity, trauma, challenge or threat.69–71 Sometimes casually referred to as bounce-back ability or ‘ordinary magic’,72 the question becomes not whether one is resilient but rather how the capacities that occasion resilient responses are created and maintained. In this respect, research highlights psychological features such as self-belief, tolerance of solitude, optimism, coping strategies and perseverance among others73, 74 but also the conditions required for their production such as a minimum of one person who believes in you, experiences that confirm competence and promote self-esteem, expansion of opportunities for new experiences, safe risk taking environments, experiences of successful problem solving, positive recognition by others.75 Conversely, social conditions may inhibit and disallow the creation of resilient features for reasons that lie entirely outside of the agency of the individual such as lack of economic opportunity, discrimination and stigma. The supported film and photography training the young people experienced combined with the opportunity to develop their own imaginative work essentially created the conditions in which significant resilience enhancing capacities could be identified, discussed and grow.

First, the young people were afforded multiple experiences of success built on recognition of inherent strength—visual embodied practices. This success was performed not just through the practices of filmmaking/photography but also through the group environment of multiple viewing and the peer critiquing of work in a social context in which shared meanings of what was good and what needed refinement were built. Many of these children had few experiences of ‘mastery’ in their lives as many were from families with little or no mutual communication (if they had a family) and school for many was a daily struggle with literacy and understanding. Taking photos and making films were something they excelled at and furthermore were *recognised* to be good at it both by their peers and the outsiders who were the project team. This external recognition continues. Their work has been seen in an exhibition as part of the 20th anniversary celebrations of the Centre for Deaf Studies at the University of the Witwatersrand and is the subject of international exhibitions, including the Children’s Museum of the Arts, New York (2020), the KwaZulu Natal Society of Arts, Durban (2019), and the Whitworth Gallery, Manchester (2020).

Second, for the deaf young people involved there was a sustained agentic power in the opportunity to shape a version of their life to themselves and others in the films and photographs produced, including their control over content, composition and production. The compositions were volitional acts but also ones in which the young people experienced the possibilities of agentic choices of representation of self and others. There were numerous examples of participants discovering how they might shape the portrayal of another in body, location and dialogue as well as discovery of how they were seen by others and therefore choices they might have in how to represent the self. This kind of agency was extended in later phases of the work to explore personal actions to promote personal safety ranging from what it might mean to embody confidence on the street rather than vulnerability for example. (The work on safeguarding is the subject of a separate forthcoming paper.).

Third, a component of the work undertaken was focused on aspiration. A hopeful but critically realistic outlook to the future is also a component of resilience. The young people had the opportunity to explore what they thought was possible for their lives in the future and think about the potential boundaries on those aspirations. Envisaging futures presupposes to some extent having the language in which to express those futures which is a major constraint for some deaf children with poorly developed language. However, the evocations of futures through filmic methods provide a means of bypassing such constraints as the visual imagination does not presuppose the necessity of language first for its release. The older participants in particular used some of the scenarios created in their later short film productions to confront common realities that impacted on hopes. Examples included being thought stupid because they could not talk when interviewing for a job and the not unusual experience of rape and sexual assault in school environments. Other films encapsulated finding a partner, becoming a school teacher and other successfully accomplished aspirations. The group storyboarding of the films, acting and showing of the finished article provided a forum in which aspirations might be articulated, examined and refined. Some of the young people went on to use their skills to film an annual deaf school gala at the request of the head teacher thus being seen by many other children, both deaf and hearing, as skilled users of a camera which potentially opened the doors to a new career.

## Conclusion

It has been remarked that in the history of humankind the first division of labour was the separation of the mind from the hand as the artisans and the thinkers took different social routes.76 Sign language peoples challenge this and other assumptions of separations of thinking from doing because, as we have demonstrated, the mind cannot be separated from the embodied practices of language and the occlusion of language with speech is disrupted as instead vision becomes the source of telling and understanding. Our initial distinction between the ontic and the ontological in conceptualising the visual orientations of deaf people has been refined by examination of the nature of the material images produced and their relationship with the context in which deaf ontologies are produced, enacted and developed. To this end, we have offered an emphasis on the affordances and constraints of *ontological potentialities* to any analysis of deaf worldmaking that might be expressed in visual terms. We have firmly linked deaf ontologies with the material and societal conditions which serve to (re)create the subjectivities that deaf young people in South Africa have to negotiate on an ongoing basis. Consequently, visual methods and praxis are offered not just as a means to respond to inherent visual strengths associated with ontological orientations of deaf young people but rather as a creative and disruptive technology through which to challenge the social production of constraint, limitations and low expectations imposed on deaf young people. We frame the photographic products as material means to write into reality deaf young people’s worldmaking in an otherwise orthographically dominant and orthographically shaped means of cultural production. In this respect, the etymological origins of ‘photographic’—writing with light—are most apt.

Our examination of deaf young people’s engagement and creativity in filmmaking has led us to propose the notion of *corporeally attenuated observation* which grew out of many examples in the young people’s work of imagistic and non-symbolic modes of thinking. In these, image and sensation, the embodied and the emotional, the morphological and the referential, the bodily and the communicative, were elided in choice of the visual image, object placement, camera movement and composition. Although not unique to deaf people, the young people in our study with little or no prior experience of filmmaking displayed exceptional creative abilities in this regard. In terms of expanding our understanding of deaf young people’s worldmaking, it was additionally a distinctive example of the inseparability of subjugated knowledges and practices in how we understand such worldmaking. Importantly, these arguments are not disconnected with the project’s initial resilience-enhancing objectives. The process and praxis of photography and filmmaking have been revealed as a powerful process through which those deaf young people who participated have experienced an asset-based process which enabled their own and others’ recognition of their abilities, a means of communication and expression of alterity that positively reframes and challenges the material, attitudinal and social conditions that surround them and the mechanism by which subjugated knowledges can be enacted and instantiated at one and the same time. The world is indeed revealed as patiently waiting for our senses to grow sharper…

## Notes

1. Our argument and the subject of this paper does not refer to deaf blind people whose communicative experiences and perception of the world are different than those of d/Deaf people and deserving of separate consideration.

2. D. Bavelier et al. (2000). “Visual Attention to the Periphery is Enhanced in Congenitally Deaf Individuals.” *The Journal of Neuroscience* 20, no. 17: RC 93.

3. J.B Jepsen et al. (2015). *Sign Languages of the World: A Comparative Handbook*. Berlin: De Gruyter Mouton.

4. K Emmorey and S M Kosslyn (1996) “Enhanced Image Generation Abilities in Deaf Signers: A Right Hemisphere Effect.” *Brain and Cognition* 32, no.1: 28–44.

5. M Marschark and P.C. Hauser (2008). “Cognitive underpinning of learning by deaf and hard-of- hearing students: Differences, diversity and directions.” In *Deaf Cognition: Foundations and Outcomes* edited by M Marshark and P.C. Hauser, 3–23. New York, NY: Oxford University Press, 2008.

6. Velia Cardin et al. (2013) “Dissociating Cognitive and Sensory Neural Plasticity in Human Superior Temporal Cortex.” *Nature Communications* 4, no.1473. DOI: 10.1038/ncomms2463

7. B Bahan (2008). “Upon the Formation of a Visual Variety of the Human Race.” In *Open Your Eyes: Deaf Studies Talking*, edited by H.D.L. Bauman, 83-99. Minneapolis, MN: University of Minnesota Press.

8. B Bahan (2009) “Sensory Orientation”. *Deaf Studies Digital Journal*. Retrieved September 19, 2015, from http://dsdj.gallaudet.edu/index.php?view=entry&issue=1&entry_id=48

9. H Lane, R.C. Pillard, and U. Hedberg (2010). *People of the Eye: Deaf Ethnicity and Ancestry*. New York, NY: Oxford University Press.

10. G De Clerck (2017). *Deaf Epistemologies, Identity and Learning: A Comparative Perspective*. Washington DC: Gallaudet University Press.

11. M Friedner (2016) “Understanding and Not-Understanding: What Do Epistemologies and Ontologies Do in Deaf Worlds?” *Sign Language Studies* 16, no. 2: 184-203. https://doi.org/10.1353/sls.2016.0001

12. Martin Heidegger (1927). *Being and Time,* trans. J. Macquarrie, and E. Robinson. New York: Harper & Row.

13. Kit Fine (1995)Ontological dependence. *Proceedings of the Aristotelian Society. New Series* :269-290. https://www.jstor.org/stable/4545221

14. Hannah Anglin-Jaffe (2011), “Sign, Play and Disruption: Derridean Theory and Sign Language.” *Culture, Theory and Critique* 52, no.1: 29-44.

15. Alys Young, Rosemary Oram, and Jemina Napier (2019) “Hearing People Perceiving Deaf people Through Sign Language Interpreters at Work: on the Loss of Self Through Interpreted Communication. *Journal of Applied Communication Research* 47, no.1:90-110. https://doi.org/10.1080/00909882.2019.1574018

16. J Derrida (1976). *Of grammatology*. (G. Spivak, Trans. 1979). Baltimore, MD: Johns Hopkins University Press.

17. A.G Bell (1884). *Memoir on the Formation of a Deaf Variety of the Human Race*. USA: National Academy of Sciences.

18. H. D. L Bauman and J. J Murray (2010). “Deaf Studies in the 21st Century: ‘Deaf-gain’ and the Future of Human Diversity.” In edited by M. Marschark & P. Spencer, The Oxford Handbook of Deaf Studies, Language, and Education, 196-225. New York: Oxford University Press.

19. D Bauman and J. J Murray (2014). *Deaf Gain: Raising the stakes for human diversity*. Minneapolis: University of Minnesota Press.

20. Alys Young, Lorraine Green, and Katherine Rogers (2008) “Resilience and Deaf Children: A Literature Review.” *Deafness & Education International* 10, no.1: 40-55, DOI:10.1179/146431508790559850

21. KA Shogren et al. (2015) “Causal Agency Theory: Reconceptualizing a Functional Model of Self-Determination.” *Education and Training in Autism and Developmental Disabilities*. 50, no.3: 251–263

22. UNICEF, 2012. *Violence against children in South Africa*. http://www.cjcp.org.za/uploads/2/7/8/4/27845461/vac_final_summary_low_res.pdf

23. Claudine Störbeck and Alys Young (2016) “The HI HOPES Data Set of Deaf Children Under the Age of 6 in South Africa: Maternal Suspicion, Age of Identification and Newborn Hearing Screening.” *BMC Pediatrics* 16, no.45. doi: 10.1186/s12887-016-0574-1

24. JCIH (2007) “Position Statement: Principles and Guidelines for Early Hearing Detection and Intervention Programs.” *Pediatrics* 120, no.4: 898–921.

25. National Assembly of South Africa. “Written reply to parliamentary question 829”, published 16/03/2018.

26. M Bachelor (2018) Personal communication from the Principal of MCK School, Lenasia, South Africa. https://www.mck.org.za/about/

27. OECD (2018). “DAC list of ODA recipients”. Retrieved June 7, 2019, from http://www.oecd.org/dac/financing-sustainable-development/development-finance-standards/daclist.htm

28. Africa Check (2018). Retrieved June 7, 2019, from https://africacheck.org/factsheets/factsheet-south-africas-official-poverty-numbers/

29. In referring to racial classifications used in documents pertaining to South Africa’s population such as this one (Africa Check, 2018) which derives its figures from South Africa’s official government statistics “Stats SA” (http://www.statssa.gov.za/), we acknowledge that there is ongoing debate within South Africa about the validity of the racial classifications officially used given the complexity of population’s cultural/linguistic characteristics and heritage and the extent to which race as a primary marker of ethnicity should be used in a post-apartheid era. Classifications of Black African, Coloured, Asian/Indian and White derive directly from the Apartheid era during which perception and classification of race was a powerful instrument of oppression (https://www.thoughtco.com/racial-classification-under-apartheid-43430)

30. SAIRR (SA Inst of Race Relations). *SA Survey 2010/2011. Health and Welfare*. http://irr.org.za/reports-and-publications/south-africa-survey/south-africasurvey-online-2010-2011/health-and-welfare

31. R Swannack (2018). “Deaf Futures: Challenges in Accessing Health Services.” Unpublished Master in Social Science in Anthropology dissertation, University of Cape Town.

32. Ross E Mitchell, Michael A Karchmer, and M.A. Karchmer (2004) “Chasing the mythical ten percent: parental hearing status of deaf and hard of hearing students in the United States.” *Sign Languages Studies*, no.4: 138-163.

33. Savo Heleta (2016) “Decolonisation of higher education: dismantling epistemic violence and Eurocentrism in South Africa.” *Transformation in Higher Education*, no.1.

34. E Oliver and W.H. Oliver (2017) “The colonisation of South Africa: a unique case.” *HTS Theological Studies*, no.3: 1-8.

35. P Ladd (2003). *Understanding Deaf Culture: In Search of Deafhood*. Clevedon: Multilingual Matters Ltd.

36. M Banks and H Morphy (1999). *Rethinking Visual Anthropology*. Yale University Press.

37. John Collier and M. Collier (1986). *Visual Anthropology: Photography as a Research Method*. Albuquerque: University of New Mexico Press.

38. S Pink (2006). *The Future of Visual Anthropology*. London: Routledge.

39. A Kusters, S Sahasrabudhe, and A Gopalakrishnan (2016). *A Reflexive Report on Filmmaking within a Linguistic Ethnography with Deaf and Hearing People in Mumbai*. MMG Working Paper 16-04.

40. D O’Brien (2013). “Visual Research with Young d/Deaf people – An Investigation of the Transitional Experiences of d/Deaf Young People from Mainstream Schools using Auto-driven Photo-elicitation Interviews.” *Graduate Journal of Social Science* 10, no.2: 152- 175.

41. D O’Brien and A Kusters (2017). “Visual methods in Deaf Studies: Using photography and filmmaking in research with deaf people” In *Innovations in Deaf Studies: The Role of Deaf Scholars* edited by, A Kusters, M De Meulder and D O’Brien. New York: Oxford University Press.

42. E. Thoutenhoofd (1998) “Method in a Photographic Enquiry of being Deaf.” *Sociological Research Online* 3, no.2: 1-18. http://www.socresonline.org.uk/3/2/2.html

43. J Rouch (2003). “Ciné-Ethnography”, edited and translated by Steven Feld. University of Minnesota Press.

44. S Langer (1979). *Philosophy in a new key: A study in the symbolism of reason, rite and art*. Cambridge, MA: Harvard University Press.

45. Anna Grimshaw (2001) “Teaching visual anthropology. Notes from the field.” *Ethnos*, no.66: 237-258.

46. J Barzun (1983). *A Stroll with William James*. New York: Harper & Row.

47. M Foucault (1982) “The Subject and Power.” *Critical Inquiry* 8, no. 4: 777-795.

48. E. A. Povinelli (2012) “The Will to be Otherwise / The Effort of Endurance.” *South Atlantic Quarterly* 111, no. 3: 453–475. https://doi.org/10.1215/00382876-1596236

49. S Worth and J Adair (1972). *Through Navajo Eyes: An Exploration in Film Communication and Anthropology*. Bloomington: Indiana University Press.

50. S Worth (1977). “Towards an Ethnographic Semiotic.” In Proceedings UNESCO 1978. Paris: Unpublished. http://www.unipune.ac.in/snc/cssh/HistorySociology/BDOCUMENTSONHISTORYOFSOCIOLGYINRESTOFTHEWORLD/7DEBATESONETHNOGRAPHYSOCIOLOGYANDANTHROPOLOGYOUTSIDEINDIA/B701.pdf.

51. Sarah Elder and S Elder (1995) “Collaborative Filmmaking: An Open Space for Making Meaning, A Moral Ground for Ethnographic Film.” *Visual Anthropology Review*, no. 11: 94–101. doi:10.1525/var.1995.11.2.94.

52. D Boyer (2006). “Turner’s Anthropology of Media and Its Legacies.” *Critique of Anthropology* 26, no,1: 47–60. doi:10.1177/0308275X06061483.

53. Terence Turner (1991) “ Social Dynamics of Kayapo Video Media in an Indigenous Society: The Cultural Meaning and the Personal Politics of Video-Making in Kayapo Communities.” *Visual Anthropology Review*, no.7: 68–76.

54. E Michaels (1986). *The Aboriginal invention of television in Central Australia, 1982–86*. Canberra: Australian Institute of Aboriginal Studies.

55. Onno Crasborn (2010) “What Does Informed Consent Mean in the Internet Age? Publishing Sign Language Corpora as Open Content.” *Sign Language Studies* 10, no.2: 276-290.

56. D O'Brien and S Emery (2013). “The Role of the Intellectual in Minority Group Studies: Reflections on Deaf Studies in Social and Political Contexts.” *Qualitative Inquiry* 20, no.1: 27-36.

57. A Young and B Temple (2014). *Approaches to Social Research: The Case of Deaf Studies*. New York: Oxford University Press.

58. M Warner, 1976. *Alone of all her Sex: The Myth and Cult of the Virgin Mary*. Oxford: Oxford University Press.

59. L Davis (1995). *Enforcing Normalcy: Disability, Deafness and the Body*. London: Verso.

60. Ann Cunliffe and Chris Coupland (2012) “From Hero to Villain to Hero: Making Experience Sensible Through Embodied Narrative Sensemaking.” *Human Relations*, no. 65: 63-88.

61. M Merleau-Ponty, 1962/2004. *Phenomenology of Perception* (Smith C, trans.). London and New York: Routledge, 1962/2004.

62. Katherine D Rogers et al. (2013) “The challenges of translating the clinical outcomes in routine evaluation-outcome measure (CORE-OM) into British Sign Language.” *Journal of Deaf Studies and Deaf Education*, no. 18: 287-298. doi: 10.1093/deafed/ent002.

63. S Beckett (1984). *Collected Shorter Plays of Samuel Beckett*. London: Faber and Faber.

64. Andrew Feenberg and A Feenberg (2000). “The ontic and the ontological in Heidegger's philosophy of technology: response to Thomson.” *Inquiry*, no.43: 445-450.

65. E Stern (2015) “A Grasp on Human Thinking.” *Nature* 524: 158-159.

66. J Butler (1999). *Gender Trouble: Feminism and the Subversion of Identity*. New York: Routledge.

67. R M Calderon and T. Greenberg (2000) “Challenges to parents and professionals in supporting socioemotional development in deaf children”. In *The Deaf Child in the Family and at School,* edited by P.E. Spencer, C. Erting, M. Marschark. New Jersey: Lawrence Erlbaum Associates.

68. M.T Greenberg and C.A. Kusche (1993) *Promoting Emotional and Social Development in Deaf Children: the PATHS Project*. Seattle WA: University of Washington Press.

69. Michael Rutter and M Rutter (1987) “Psychosocial Resilience and Protective Mechanisms.” *American Journal of Orthopsychiatry* 57: 316–331.

70. M Rutter (1993) “Resilience: Some Conceptual Considerations.” *Journal of Adolescent Health* 14: 626–631.

71. M Ungar (2004) “A Constructionist Discourse on Resilience: Multiple Contexts, Multiple Realities Amongst At-risk Children and Youth”. *Youth and Society* 35, no.3: 341–365.

72. A S Masten (2001) “Ordinary Magic: Resilience Processes in Development.” *American Psychologist* 56, no. 3: 227-238. DOI: 10.1037//0003-066X.56.3.227

73. Lanae Valentine and Leslie L. Feinauer (1993) “Resilience Factors Associated with Female Survivors of Childhood Sexual Abuse.” *American Journal of Family Therapy* 21, no.3: 216–224.

74. Trine Waaktaar et al. (2004) “How Can Young People’s Resilience Be Enhanced? Experience from a Clinical Intervention Project.” *Clinical Child Psychology and Psychiatry* 9, no. 2: 167–183.

75. R Gilligan (1998) “Enhancing the Resilience of Children and Young People in Public Care by Mentoring their Talent and Interests.” *Child and Family Social Work*, no. 4: 187–196.

76. M Pasquinelli (2018). Artificial intelligence or the demonization of mental labor. In *The Demon’s Brain* edited by A Polska, 50-54. Berlin: National Gallery.
